# Central Aortic Cannula Disruption Following Left Atrial Myxoma Excision

**DOI:** 10.7759/cureus.41908

**Published:** 2023-07-14

**Authors:** Nitin Kumar Kashyap, Pranay Mehsare, Gaind Saurabh, Nirupam Chakraborty, Minal Wasnik

**Affiliations:** 1 Cardiothoracic Surgery, All India Institute of Medical Sciences, Raipur, Raipur, IND; 2 Transfusion Medicine & Blood Bank, All India Institute of Medical Sciences, Raipur, Raipur, IND

**Keywords:** cardiotomy suction, metallic tip of cannula, heart-lung machine, aortic cannula breakage, cardiopulmonary bypass, myxoma, cell saver, blood salvage, cpb, aortic cannula disruption

## Abstract

Central aortic cannulation is used to give oxygenated blood to the patient through a heart-lung machine. Central aortic cannula disruption during cardiopulmonary bypass (CPB) is a rare complication. This could result in aortic dissection, extensive tears, bleeding, posterior aortic wall injury, oesophageal trauma, and cardiac arrest. We are reporting a central aortic cannula disruption during a left atrium (LA) myxoma excision in which the metal tip part of the cannula detached from its body, resulting in massive blood loss. The intraoperative blood salvage technique was used to maintain hemodynamics during surgery. Pre-procedural visual inspection of all cardiac consumables, including cannula, should be performed to eliminate this complication. All surgical team members should be observant to avoid such complications.

## Introduction

The aortic cannula is used to connect the “arterial limb” of the cardiopulmonary bypass (CPB) circuit to the patient so that oxygenated blood from the heart-lung machine can be directly delivered into the patient’s arterial system [1]. Aortic cannulation is one of the significant steps in cardiac surgery. Many complications can occur during aortic cannulation like aortic dissection, extensive tear, bleeding, posterior wall injury, redundant trauma to the esophagus, and even cardiac arrest. Disruption of the aortic cannula is a rare but potentially fatal complication.

We are reporting a case of central aortic cannula disruption during left atrial myxoma excision. Surgeons and perfusionists should always be alert to catch up on minor manufacturing defects in these disposable items. They can ensure safety by including these items through precheck to avoid complications during operative procedures [2]. 

This article was previously posted to the Research Square preprint server on June 21, 2021.

## Case presentation

A 45-year-old male presented to the outpatient department with the chief complaint of palpitation for four months. On general physical examination, BP was 120/60 mm/Hg, pulse rate was 74 beats/minute, respiratory rate was 20/minute, and saturation of peripheral oxygen (Spo2) on room air was 97%. Systemic examination revealed normal heart sounds with a mid-diastolic murmur. Electrocardiogram (ECG) showed a regular sinus rhythm. Pre-operative trans-thoracic echocardiography revealed an intra-cardiac tumor attached to the interatrial septum, with a mild grade of mitral regurgitation.

Clinical presentation and echocardiography favor the diagnosis of left atrial myxoma. The patient was planned for left atrial myxoma excision under CPB. CPB was initiated as per conventional procedure by bicaval venous drainage, and arterial return was maintained through ascending aortic cannulation with 22 Fr, new metal tip aortic cannula. Topical cooling, along with mild hypothermia, was achieved up to 32 degree Celsius. The cross-clamp was applied, and cardioplegic arrest was accomplished with an antegrade cold del Nido cardioplegia solution. The tumor was approached through the right atrium, the intra-atrial septum was incised, and excision of the tumor was performed. The interatrial septum and right atrium were closed, and the cross-clamp was released after good de-airing of the heart. The electrical activity of the heart regained spontaneously. During the termination of CPB, the pleural cavity and mediastinal space were filled with bright red arterial blood. A sudden loss in venous reservoir volume in the heart-lung machine was noted as most of the blood volume was present in these cavities. Hence, intraoperative blood salvage was performed through cardiotomy suction and replaced.

**Figure 1:**
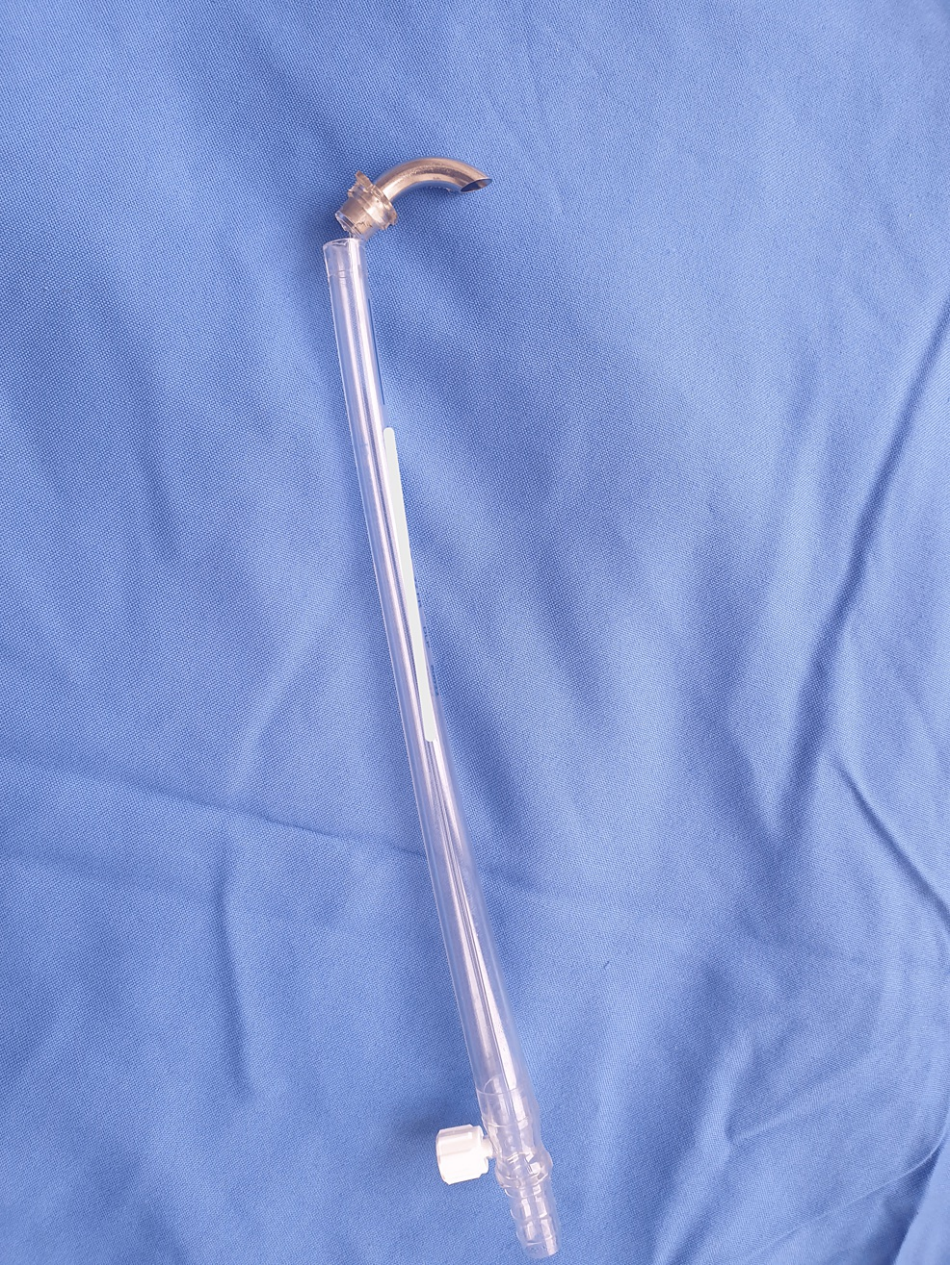
The metal tip part of the aortic cannula got detached partially along with its flange part

On thorough inspection, the aortic cannula was found to be broken from its flange side, resulting in the leakage of arterial blood into the pleural cavity and mediastinal space and a very high chance of air embolism. Hence, The patient was put on the Trendelenburg position, and the pump flow was gradually reduced and weaned off bypass with low systemic perfusion pressure. The aortic cannula was removed as early as possible. Immediately after decannulation, the metal tip of the aortic cannula detached partially along with its flange part and was about to separate from its body (Figure 1). The operative procedure was completed successfully. The patient was extubated on the same day in the intensive care unit (ICU) without any neurological deficit.

Postoperative echocardiography did not reveal any residual mass inside the left atrium or mitral valve regurgitation. Both ventricular functions were normal. The patient was discharged on the seventh postoperative day. He had no complaints on his regular follow-up after six months.

## Discussion

Major surgical intraoperative bleeding during cardiac surgery is a dreaded complication which results in significant morbidity and mortality [3]. Aortic cannula disruption is one such rare cause. The aortic cross-clamp had already been released during the operation, so our earliest attempt was to decannulate the patient and wean him off from CPB. In a case reported by Scheld et al., the metal tip part was wholly disconnected from the body and later discovered at the bifurcation of the aorta during an on-pump coronary artery bypass graft (CABG) [4].

The disruption could be due to poor junctional attachment between the metal tip and body during manufacture or tearing of the cannula with the snugger by heavy silk, which may cause a pinching effect of the cannula leading to disruption. During cardiac surgery, the patient is submitted for moderate hypothermia and then rewarmed again. This temperature change may cause an impact on the expansion and contraction of the cannula material, which may or may not be significant. The metallic tip and the cannula tubing are made up of different substances and varied expansion coefficients. This may lead to a considerable size change, causing the cannula tip to loosen from the tubings and its possible disconnection. However, in the rare possibility of this occurrence, it is less likely to be one of the causes of the same [2]. We feel the poor attachment of the metal tip with the body during manufacturing was the possible cause of complication in our case.

The salvaged blood from cardiotomy suction was re-transfused through the central venous catheter line. One study has suggested using intraoperative cell salvage and autologous blood transfusion as an important method of blood conservation to reduce the need for allogeneic blood transfusion and its associated complications [5]. Cell saver facility was unavailable in our institute, so we had to salvage blood through cardiotomy suction. The situation in which cannula disruption occurs during the cross-clamp period can be more dangerous. During the cross-clamp period, the aortic cannula is the only way to oxygenate the patient during extracorporeal circulation [1]. Management involves prompt temporary cessation of CPB with emergency recannulation. During emergency recannulation, due to low pressure, the aorta tends to collapse, which will also carry a greater risk of damaging the back wall of the aorta [6]. If recannulation of the ascending aorta is not feasible or no space is available due to multiple procedures involving the ascending aorta, alternate femoral or iliac arterial cannulation strategy is indicated [7,8]. We recommend a pre-inspection of all such cannula and other cardiac consumables before considering them for use.

## Conclusions

Aortic cannula disruption is a rare complication that can be lethal to the patient if not managed promptly. We should not reuse cannulas in all circumstances. Avoiding significant temperature variations and using sutures for snugger ties is not a practical and sure-shot measure to prevent such incidence. Before cannulation, proper visual inspection of all cannula by team members is crucial to eliminate this complication. During cardiac surgery, everyone on the team must be cautious and observant to avoid such dangerous complications.

## References

[1] Chilton V, Klein A (2009). .Equipment and monitoring. Cardiopulmonary Bypass, 1st edition.

[2] Gargava A, Sarkar M, Umbarkar S, Shringarpure A (2020). Aortic cannula tip dislodgement: a rare complication. Ann Card Anaesth.

[3] Petrou A, Tzimas P, Siminelakis S (2016). Massive bleeding in cardiac surgery. Definitions, predictors and challenges. Hippokratia.

[4] Scheld HH, Görlach G (1985). Disconnection of the metallic tip of an aortic cannula during cardiopulmonary bypass. Thorac Cardiovasc Surg.

[5] Ashworth A, Klein AA (2010). Cell salvage as part of a blood conservation strategy in anaesthesia. Br J Anaesth.

[6] Eugene A. Hessel II (2012). Circuitry and cannulation techniques. Cardiopulmonary Bypass: Principles and Practice, 3rd edition.

[7] Mills NL, Everson CT (1991). Atherosclerosis of the ascending aorta and coronary artery bypass. Pathology, clinical correlates, and operative management. J Thorac Cardiovasc Surg.

[8] Wareing TH, Davila-Roman VG, Barzilai B, Murphy SF, Kouchoukos NT (1992). Management of the severely atherosclerotic ascending aorta during cardiac operations. A strategy for detection and treatment. J Thorac Cardiovasc Surg.

